# The Next Frontier of Environmental Unknowns: Substances of Unknown or Variable Composition, Complex Reaction Products, or Biological Materials (UVCBs)

**DOI:** 10.1021/acs.est.2c00321

**Published:** 2022-05-09

**Authors:** Adelene Lai, Alex M. Clark, Beate I. Escher, Marc Fernandez, Leah R. McEwen, Zhenyu Tian, Zhanyun Wang, Emma L. Schymanski

**Affiliations:** † Luxembourg Centre for Systems Biomedicine (LCSB), University of Luxembourg, 6 avenue du Swing, 4367 Belvaux, Luxembourg; ‡ Institute for Inorganic and Analytical Chemistry, Friedrich-Schiller University, Lessing Strasse 8, 07743 Jena, Germany; § Collaborative Drug Discovery Inc., 1633 Bayshore Highway, Suite 342, Burlingame, California 94010, United States; ∥ Helmholtz Centre for Environmental Research GmbH—UFZ, Permoserstraße 15, 04318 Leipzig, Germany; ⊥ Environmental Toxicology, Center for Applied Geosciences, Eberhard Karls University Tübingen, 72076 Tübingen, Germany; # Environment and Climate Change Canada, 401 Burrard Street, Vancouver, British Columbia V6C 3R2, Canada; ∇ Cornell University, Ithaca, New York 14850, United States; ○ International Union of Pure and Applied Chemistry, Research Triangle Park, North Carolina 27709, United States; ◆ Department of Chemistry and Chemical Biology, Department of Marine and Environmental Sciences, Northeastern University, Boston, Massachusetts 02115, United States; ¶ Empa—Swiss Federal Laboratories for Materials Science and Technology, Technology and Society Laboratory, Lerchenfeldstrasse 5, 9014 St. Gallen, Switzerland; ▲ Chair of Ecological Systems Design, Institute of Environmental Engineering, ETH Zürich, 8093 Zürich, Switzerland

**Keywords:** mixtures, UVCB, complex substances, testing and assessment, cheminformatics, environmental pollutants

## Abstract

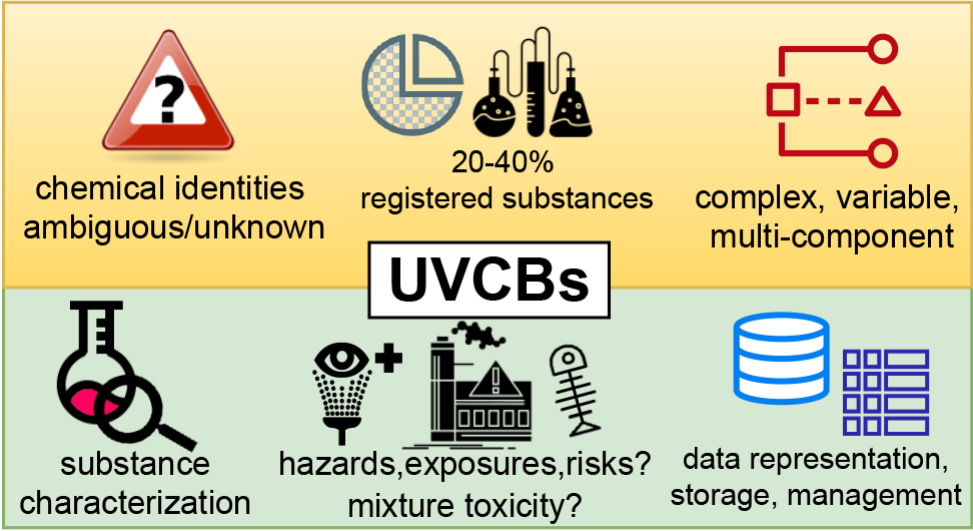

Substances of unknown or variable composition, complex reaction products, or biological materials (UVCBs) are over 70 000 “complex” chemical mixtures produced and used at significant levels worldwide. Due to their unknown or variable composition, applying chemical assessments originally developed for individual compounds to UVCBs is challenging, which impedes sound management of these substances. Across the analytical sciences, toxicology, cheminformatics, and regulatory practice, new approaches addressing specific aspects of UVCB assessment are being developed, albeit in a fragmented manner. This review attempts to convey the “big picture” of the state of the art in dealing with UVCBs by holistically examining UVCB characterization and chemical identity representation, as well as hazard, exposure, and risk assessment. Overall, information gaps on chemical identities underpin the fundamental challenges concerning UVCBs, and better reporting and substance characterization efforts are needed to support subsequent chemical assessments. To this end, an information level scheme for improved UVCB data collection and management within databases is proposed. The development of UVCB testing shows early progress, in line with three main methods: whole substance, known constituents, and fraction profiling. For toxicity assessment, one option is a whole-mixture testing approach. If the identities of (many) constituents are known, grouping, read across, and mixture toxicity modeling represent complementary approaches to overcome data gaps in toxicity assessment. This review highlights continued needs for concerted efforts from all stakeholders to ensure proper assessment and sound management of UVCBs.

## 1 Introduction

Anthropogenic chemical pollution is pervasive and has been found in multiple environments,^1−5^ animals,^6−9^ and humans^10−14^ worldwide, with at least 16% of global premature deaths attributed to diseases caused by pollution.^15^ Chemical pollutants originate from the production, use, and disposal of diverse chemical products. The most familiar and well-studied are single chemical compounds, but these form only a part of the bigger picture of chemical pollution. In practice, many pollutants come from chemical products consisting of mixtures. While some of these mixtures are well-defined, many are poorly characterized or contain constituents with unknown or variable chemical identities, and they are classified as substances of unknown or variable composition, complex reaction products, or biological materials (UVCBs).

UVCBs are considered chemical substances within multiple legal frameworks,^16−18^ and thus they are subject to various registration, hazard evaluation, and risk assessment requirements. UVCBs can be found everywhere: within detergents, fragrances, and personal care products, and even within fuel and starting materials for chemical manufacturing. A broad range of substances are considered UVCBs, e.g., those of natural origin such as petroleum fractions and essential oils, synthetic products such as technical mixtures of specialty copolymers, and reaction products such as medium-chain chlorinated paraffins (MCCPs; CASRN 85535-85-9) and substances such as “Rape oil, reaction products with diethylenetriamine” (CASRN 91081-13-9; all UVCBs mentioned in this review are detailed in Table S1). As such, UVCBs may contain structurally similar (e.g., isomers, homologues, congeners), or entirely dissimilar chemical constituents. Variations in their composition may arise from fluctuations in production processes, starting materials, or the presence of transformation products formed from spontaneous reactions.

UVCBs are highly prevalent on the global market: 20–40% of chemicals registered in the European Union and in the United States comprise UVCBs.^19−21^ A recent global inventory found over 70 000 UVCBs and polymers within over 235 000 registered chemicals with Chemical Abstracts Service Registry Numbers (CASRNs).^22^ Additionally, many UVCBs are produced and used at high volumes globally. Annual production of MCCPs in China alone was estimated to be 600 000 t in 2013,^23^ and 1027 million metric tons of petroleum substances were manufactured or imported into the European Union in 2018.^24^

Given their significant proportion within chemical registries, high production volumes, and wide usage patterns, UVCBs are highly environmentally relevant. While certain UVCBs such as linear alkylbenzenesulfonate surfactants were found at high intensity in wastewater,^25^ the chemical identities of most UVCBs remain unknown or poorly characterized. These critical information gaps limit their detection and identification in the environment and biota, and hinder assessment of their hazards and risks, particularly as most existing testing methods were originally designed for discrete compounds. Meanwhile, current information systems and cheminformatic representations are ill-equipped to store, index, and retrieve information on UVCBs from databases. Consequently, UVCBs are commonly omitted from scientific studies for the sake of simplicity,^26−28^ and regulators around the world face challenges in assessing and managing their environmental and health risks.^29^

Rather than tackle UVCBs as a substance *class*, previous reviews focused on specific substances using a single disciplinary lens: e.g., analytical characterization of chondroitin sulfate^30^ and surfactants,^31^ health assessment of endocrine-disrupting chemicals in oil and natural gas,^32^ environmental risks of MCCPs,^23^ and toxicology and epidemiology of bentonite.^33^ The sole review that tackles UVCBs as a substance class only addresses aspects of its risk assessment.^29^ Meanwhile, reviews on chemical mixtures typically mention UVCBs only superficially^34,35^ or do not explicitly address them at all.^36,37^

In this review, UVCBs are treated as a substance class as a means of addressing common challenges across UVCBs from the perspectives of cheminformatics, analytical chemistry, toxicology, and regulatory science. This review aims to (1) provide an overview of methodological developments for addressing UVCBs across the different domains, (2) summarize general approaches taken, (3) highlight challenges and gaps, and (4) identify further areas of research toward developing shared good practices. UVCBs warrant urgent attention from both scientific and regulatory communities, and this review aims to provide tractability in tackling this next frontier of environmental unknowns.

## 2 Characterization, Identification, and Representation of UVCBs

Meaningful structural representation of a chemical is important for connecting its detection in the environment or biota to chemicals registered on the global market and subsequent assessment of hazard, fate, exposure, and risks to human health and the environment. While chemical characterization (the process of obtaining information about a substance’s constituents and composition), identification (unambiguous and precise recognition of the same substance by all stakeholders), and representation (how a chemical’s identity is communicated) are typically clear for single compounds, they are not clear for UVCBs due to the lack of structural information available on these multiconstituent substances. Consequently, there exist challenges in chemically representing UVCBs using currently established formats: as text via its name, synonym, or description; structurally as structural diagrams, Simplified Molecular Input Line Entry System (SMILES),^38^ molecular data files such as Molfile (MOL) and Structure Data File (SDF);^39^ or by identifiers such as the International Chemical Identifier (InChI),^174^ its hashed version InChIKey, and other database or registry specific identifiers, e.g., CASRN, Distributed Structure-Searchable Toxicity Substance Identifier (DTXSID), PubChem Compound Identifier (CID), and European Community List Number (EC/List No.).

### 2.1 Current State of Available Structural Information on UVCBs in the Public Domain

The current availability of UVCB structural information has largely been determined by registration requirements. A substance is categorized as UVCB during chemical registration if it adheres to UVCB specifications, as was historically the case in the United States, where nearly 10 000 UVCBs were listed in the original Toxic Substances Control Act Inventory dating back to 1979.^40,41^ Similarly in Canada and Europe, substances are determined to be UVCBs if they meet the formal definition specified in the 1999 Canadian Environmental Protection (CEPA) Act^18^ and 2017 Registration, Evaluation, Authorization and Restriction of Chemicals (REACH) Guidance, respectively.^42^

In most cases, the initial information that can be used to identify UVCBs depends upon what registrants provide via the registration systems. For example, under EU REACH legislation, registrants can report multiple constituents, concentrations, and manufacturing process details of their UVCB within the International Uniform Chemical Information Database (IUCLID).^43^ However, not all information submitted during registration is necessarily made publicly available at a level that allows for unambiguous identification of a given UVCB.^44^ Furthermore, registration frameworks in most parts of the world tend to focus on new substances, despite the existence of many older substances with little to no available information that were already on the market before registration frameworks entered into force.

**Figure 1:**
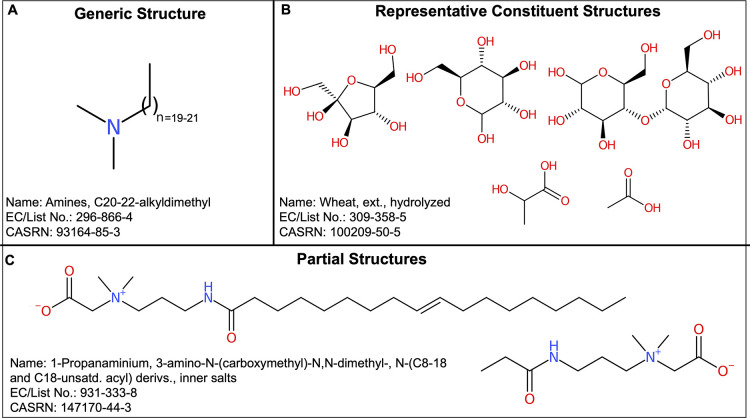
Examples of chemical structure representations for UVCBs available in REACH registration dossiers, depicted using CDK Depict.^48^

Presently, UVCBs are included in both national chemical registries and certain public databases. The major relevant databases, types of information available, and chemical representations are summarized in Table S2. Substance name is the most widely available identifier of UVCBs across all databases, and some substances have registry numbers (CASRN and/or EC No.) and/or an additional database identifier. Notably, however, substance name and identifiers for UVCBs can be ambiguous in nature.^18,42,45,46^ Complete structural diagrams are frequently optional to provide upon registration; instead, descriptive information on chemical composition, source, processing, and/or partial structural diagrams are usually accepted.^18,42,45,47^ Consequently, the vast majority of UVCBs have little to no detailed structural information (at least in the public domain), whether in the form of SMILES, InChI, structural diagram, or molecular formula. This lack of structural information is a fundamental knowledge gap concerning UVCB identities. For the few UVCBs that do have some associated structural information, their chemical representation can be single and/or multiple structure(s) as illustrated in Figure 1.

Generic structures (Figure 1A) typically encompass a range of homologues with varying chain length at a certain site or sites on the molecule. Representative constituents for UVCBs (Figure 1B) can be chosen in multiple ways, e.g., as the predominant constituent by percent composition reported in the literature, to reflect a specific end point such as toxicity, of median chain length to represent homologous constituents of varying chain lengths, or two compounds with the shortest and longest chain lengths defining the range of constituents. Representatives resulting from grouping (sections 2.2.2 and 3) or statistical selection^21,49^ are also possible. Lastly, partial structures (Figure 1C) represent one or more chemically interpretable aspects described in the substance name. Regardless of representation type, varying levels of specificity in structures (i.e., specific compound versus chemical class the compound belongs to) have been reported, resulting from being registered under the same registry number^50^ or cheminformatic import issues across various databases causing inadvertent removal of undefined substituents (“Rgroup”) or imprecise polymer (“Sgroup”) definitions.^39^

### 2.2 UVCB Characterization

UVCB characterization has been driven by increased regulatory assessments of UVCBs,^29^ developments in chemical database infrastructure,^51^ and increasing awareness of the need to identify problematic chemicals in the environment.^52^ Characterization initiatives have emerged in two main areas: cheminformatics (section 2.2.1) and analytical chemistry (section 2.2.2).

#### 2.2.1 Cheminformatics Approaches to Characterize UVCBs

##### Linking Preexisting Chemicals to UVCBs

This cheminformatics approach involves linking preexisting structures of discrete compounds to UVCBs within chemical databases. A prominent example is the CompTox Chemicals Dashboard of the United States Environmental Protection Agency (U.S. EPA),^53^ where constituents are linked to UVCBs via manually curated relationship mappings in its database. The Dashboard also includes generic (Markush) representations and so-called “Markush Children” for UVCBs with generic structures.^51^ Besides enumeration using Markush technology,^54^ molecular structure generation methods such as MOLGEN^55,56^ and simple SMILES expansion^57^ have also been explored.^58^ Another example is SciFinder’s^59^ approach: SciFinder parses a UVCB name into its individual constituents and then provides the constituent structures as output to the UVCB queried. The drawbacks of this method are that the constituents must be present in the database to begin with (or new entries need to be registered), linking is time-consuming if performed manually or more prone to errors through automatic name parsing, and final structures are not necessarily achieved. Finally, the European Chemicals Agency Database (ECHA) has a section on “Group Members” within certain Substance Infocards, which may consist of UVCB constituents (e.g., MCCP^60^), and is curated either by official sources, expert judgment, or algorithm proposed judgment. However, this grouping is intended for specific regulatory activities instead of purely linking constituents to UVCBs. Therefore, groups may also contain substances that are not constituents if these substances fall within the same regulatory group.

##### Elucidation of Chemical Structures

For certain UVCB names containing chemically interpretable parts, e.g., “Quaternary ammonium compounds, coco alkyl(2,3-dihydroxypropyl)dimethyl, 3-phosphates (esters), chlorides, sodium salts” (CASRN 173010-79-2), a trained analyst can manually elucidate (sub)structures using basic knowledge of chemical nomenclature, database searches, and depiction tools such as CDK Depict.^48^ Representative structures are chosen where necessary, and proposed structures should be chemically feasible (e.g., obey basic chemistry principles such as valence rules). In this way, the analyst effectively manually generates new structural information. However, such structure elucidation can only be validated with analytical studies^61^ and would not be applicable to UVCBs with names containing chemically uninterpretable elements such as unknown or variable starting materials, biological species, or reaction processes, e.g., “Juniper, *Juniperus mexicana*, ext., isomerized, acetylated” (CASRN 91053-33-7) or “Distillates, petroleum, steam-cracked” (CASRN 64742-91-2).

An alternative approach involves extensively searching the literature for constituent structures and their “structural variability characteristics” (e.g., physicochemical properties inferred from spectral or chromatographic data), encoding these pieces of information into formats such as generic SMILES (section 2.3), and then generating all possible constituent structures accordingly.^21,49,50^ This approach relies heavily on the availability of constituent information in the literature or from industry collaborators as well as curators’ knowledge and expert judgment to use this information, which may explain why its applicability has been limited to mostly petroleum substances so far, as expertise and information on their constituents are highly available compared to other substances.

#### 2.2.2 Analytical Chemistry Approaches to Characterize UVCBs

##### Elucidating Chemical Structures and Composition

General discussions of analytical techniques applicable to characterizing UVCBs are available elsewhere,^62,63^ but since these techniques are typically chemical class and property dependent, they must be tailored to specific UVCBs. Additionally, certain UVCBs such as petroleum substances that contain mostly hydrocarbons may be less challenging to characterize compared to UVCBs containing multiple chemical classes such as essential oils. Overall, petroleum substances appear to be the most extensively characterized UVCBs: constituent identification commonly by gas chromatography–mass spectrometry (GC–MS) and ion mobility spectrometry–mass spectrometry, and relative quantification by GC(xGC) flame ionization detection.^64−69^ Essential-oil UVCBs were characterized using low resolution GC–MS aided by available library spectra and reference standards of constituents.^70−72^ Among high resolution mass spectrometry methods, one example used five different techniques to characterize a polyhalogenated flame retardant UVCB, concluding that it is “dominated by C_18_ carbon chain lengths, substituted with 3–7 chlorine atoms and 1–3 bromine atoms on an alkane chain”.^61^ Unambiguous structural identification is often not feasible for many UVCBs such as these, as “no individual or mixed standards for [polyhalogenated (bromo-chloro) *n*-alkanes] exist”.^61^ A similarly broad characterization of chlorinated paraffins revealed the composition of the constituents’ different chain lengths.^73^ Constituent percentage compositions were also derived for organic metal salt UVCBs that required pretreatment steps for amenability to GC–MS and nuclear magnetic resonance analyses.^74^

In general, analytical characterization of UVCBs is technically challenging: first, the commercial availability of standards is limited. Petroleum UVCBs are the exception, as direct provision of standards by industry stakeholders supporting research likely contributed to intense characterization efforts over the years. Second, choosing appropriate test material may be difficult because of possible variability in substance composition. In a dossier screening study of 155 UVCB registration dossiers under REACH, 49% on average were found to have materials used for ecotoxicological end point testing that did not match the UVCB actually being registered.^75^ Biological materials in particular can have high variability. For example, chondroitin sulfate (CASRN 9007-28-7) is a polymeric UVCB isolated from animals, whose diet and lifestyle, in addition to material extraction and processing, may affect polymer composition.^30^ Likewise, a given petroleum substance produced using the same refinery process could have different compositions within or across refineries depending on the operating conditions of the processing plant and chemical composition of the crude oil feedstocks.^76^ Harmonized criteria with composition ranges^75^ for selecting UVCB reference materials could be developed, and reference material manufacturers should provide detailed characterizations of their substances that have ideally been standardized, pooled, or homogenized across multiple batches.

Selecting appropriate sample preparation, separation, and analytical methods can be especially challenging for UVCBs, as there is little, if any, prior knowledge of substance identity to guide decisions in analytical strategy. Similar to typical nontarget studies, multiple analytical techniques and an iterative approach are often needed to provide as much complementary information as possible when dealing with UVCBs.^30,61^ Ideally, both qualitative (constituent identity or bulk identities) and quantitative (constituent percent composition/concentration) characterization would be performed, highlighting the importance of both high mass resolution *and* chromatography (multidimensional if necessary for highly complex substances) in UVCB characterization. Where complete characterization is not possible, sum parameters (e.g., total carbon content, extractable organic chlorine, or total molar concentrations) can be used as intermediate descriptions.^77^

Overall, more studies and experience are needed for the analytical characterization of UVCBs, as they are so chemically diverse that there is no one method suitable for all. To date, most efforts have focused on some UVCBs of economic interest, i.e., petroleum products, and therefore other UVCBs may warrant more attention from the analytical chemistry community. A scheme prioritizing UVCBs by, e.g., known toxicity, high exposure, high production volume, or least complexity in terms of number/type of constituents may guide researchers in this area, as could the tiered approach for substance identification and characterization necessary to support ecological risk assessment that is currently under development.^29^

##### Grouping

Besides revealing compositions and information on chemical identities of individual UVCBs, analytical characterization of UVCBs enables grouping of substances and/or constituents based on common analytical features measured. Grouping helps mitigate substance complexity and multiplicity^78^ through simplifying a UVCB down to representative constituents or fractions, or a group of UVCBs to a representative UVCB, thus allowing for more efficient testing, hazard assessment, and risk assessment (section 3), and read across (i.e., using available data to predict properties of analogous substances and fill data gaps),^20,68,175^ while facilitating structural representation in databases (Figure 1B). In general, grouping should be fit for purpose as there are many strategies for and applications of grouping,^79^ such that rationale, decisions, and uncertainties should be communicated transparently.

Establishing similarity is a prerequisite for grouping. Guidance specific to oleochemicals,^80^ hydrocarbon solvents,^81^ and petroleum substances^82^ and for general chemicals^175,83^ recommends grouping based on similar structural/physicochemical properties such as the presence of common functional groups, length and branching of carbon chains, aromaticity, etc. Ion mobility and GC–MS were used to group petroleum substances on this basis, as indicated by measured features in common such as carbon chain length, double bond equivalents, and H:C ratio.^67,69^

##### Addressing Substance Variability

Analytical characterization may reveal the extent of substance variability across different samples of the same UVCB, which may affect the applicability of available data on end points, properties, and/or substance identity. For example, despite observing some variation in hydrocarbon content and composition of the solvent “White Spirit” over multiple years and geographical samples, researchers concluded that the fluctuation was so minimal that its “technical properties and toxicological effects have not substantially changed”.^84^ Conversely, insufficient similarity found among various *Gingko biloba* extracts may limit the applicability of toxicological data collected for the tested reference to untested samples.^85^

### 2.3 UVCB Identification and Representation

An appropriate representation for UVCBs is needed to facilitate unambiguous and precise identification, which in turn enables searchability. Currently, substance name is the most universally available representation across all UVCBs. However, name is problematic for searching as multiple synonyms may exist, names are sensitive to typographical errors, and they are often inconsistent across different registries/databases because there are multiple, inherently ambiguous UVCB nomenclature specifications across different jurisdictions.^18,42,45,46^ Strategies to exploit this ambiguity have been developed, e.g., using generic descriptors to mask specific chemical identities.^86,176^ Certain UVCBs such as essential oils face specific challenges: a combination of commercial, botanical, and chemical names can be used,^87^ such that the same substance can have multiple different names. Additionally, curation inaccuracies and/or quality control issues can make identification even more difficult; e.g., within the ECHA database some substances have names such as “As UVCB, this information cannot be provided” (EC No. 942-495-4), or “the substance is UVCB” (EC No. 939-895-6). After name, CASRN is the second most used representation of UVCBs, but like substance name it is imprecise and ambiguous^29,46^ and is not an open identifier. Further compounding ambiguity issues, the same combination of CASRN and substance name can be used to represent different substances.^46^

**Figure 2:**
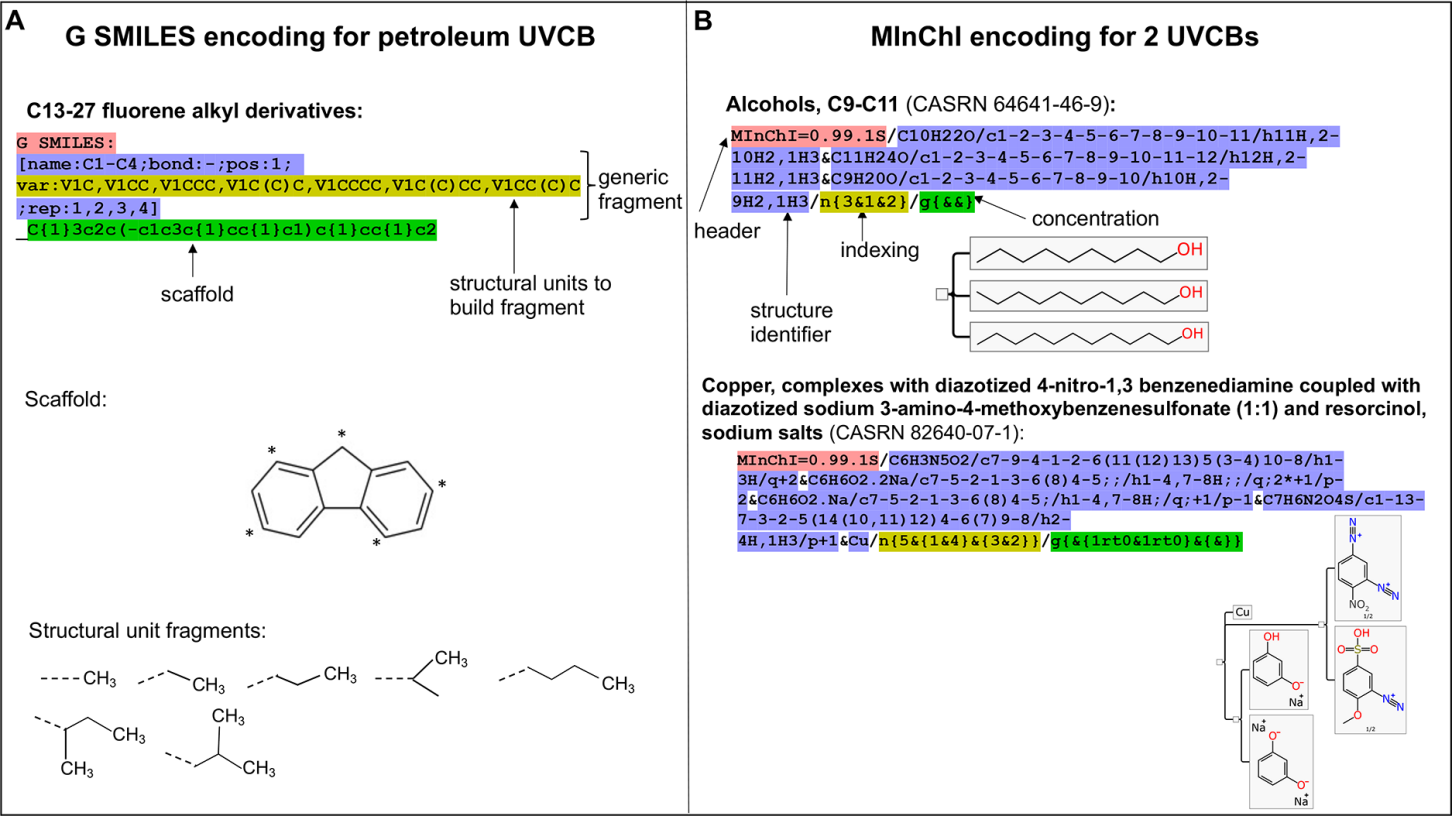
Examples of cheminformatics representations of UVCBs: (A) G SMILES. (Modified with permission from ref (50). Copyright 2015 John Wiley and Sons.) (B) Mixture InChI (MInChI).^88^ The highlighted character strings are machine-readable formats, color coded according to the different components of G SMILES and MInChI, respectively, as indicated by their labels.

For improved UVCB identification and searchability, there are currently two (complementary) alternative cheminformatics representations capable of capturing the multiconstituent, multifaceted nature of UVCB chemical systems in a machine-readable way (Figure 2). The first is generic SMILES (G SMILES), a method for structurally describing UVCBs and their variable compositions to facilitate hazard assessment via selection of representative constituents.^21,50^ G SMILES relies on a dictionary of predefined descriptors to convey generic fragment information, derived from a scaffold-fragment approach. Nonstructural descriptors such as physicochemical properties and substance formation processes are encoded in a so-called G graph. However, since this format deliberately focuses on hazard assessment, it focuses on capturing relevant structures and disregards those considered irrelevant or computationally too expensive to manage. Additionally, it may not be easily applicable to substances whose names inherently contain little chemical information and thus no structural representation as it relies on the premise of an existent molecular scaffold. The format, proposed in 2015, has yet to be formally adopted in major databases.

The second approach applies the open InChI identifier to the latest developments in mixture cheminformatics, first proposed in 2019.^89^ (Note: “mixture” is used here in the cheminformatics context of having multiple components, unrelated to the regulatory definition of mixture.) Mixture InChI (MInChI) provides a standardized definition of a given mixture that incorporates three essential properties within its notation: compound, quantity, and hierarchy. Incorporation of the InChI standard facilitates searching and linking of constituent information to public databases (e.g., PubChem). As for G SMILES, knowledge of structure is necessary to generate InChIs, which may have limited application for many UVCBs. MInChI is in active development and has an open source editor and tools to generate an upstream “Mixfile” format for additional metadata.^90^ A preliminary study has been initiated;^88^ discussions within the International Union of Pure and Applied Chemistry (IUPAC)’s MInChI project are ongoing.

#### UVCB Information Management

Improved systematic representation of UVCBs as multicomponent substances is much needed to properly manage their multifaceted information properties toward supporting chemicals assessment and monitoring. In particular, the ability to link single components and their reported characteristics back to “source” substances would support the identification and tracking of UVCBs in environmental samples—an issue that has received little concerted attention so far. Ultimately, the goal for representation of UVCBs in databases is to make them as accurate, nonambiguous, and machine readable as possible, so that entries can be easily searched, classified, and analyzed—including by constituents and between databases. Proper quality control during registration, substance representation, and database curation will be crucial to avoid “inaccurate and unrepresentative structures in databases” (as discovered for CASRN 68527-01-5).^61^

Many UVCBs are intentional mixtures of poorly defined substances (e.g., plant extracts) with well-defined and characterized adjuncts (e.g., solvents). Breaking these up into separate components hierarchically allows known properties such as toxicity to be ascribed to either individual constituents, a group thereof, or an entire substance, which would eliminate ambiguity between individual and aggregate properties and facilitate analysis at the appropriate hierarchy level.

The data structure similar to the Mixfile format described by Clark et al.^89^ could be used to achieve such systematic cheminformatic representation. Based on the principles of MInChI, the framework provided by Mixfile can be adapted to represent UVCBs at the *substance* level in terms of constituent, composition/concentration, and hierarchy. Additional metadata can be managed around these properties that facilitates cheminformatics operations and is able to handle missing or incomplete information about a given constituent. Importantly, whatever relevant chemical information available contributing to substance characterization (e.g., physicochemical properties, substance source, physical state/form, and toxicity) should be represented in a way that supports derivation of further properties via, e.g., modeling. Furthermore, especially for reaction product UVCBs, parameters such as reaction precursors, intermediates, reaction processes, and conditions of formation can be incorporated into substance characterization profiles.

**Figure 3:**
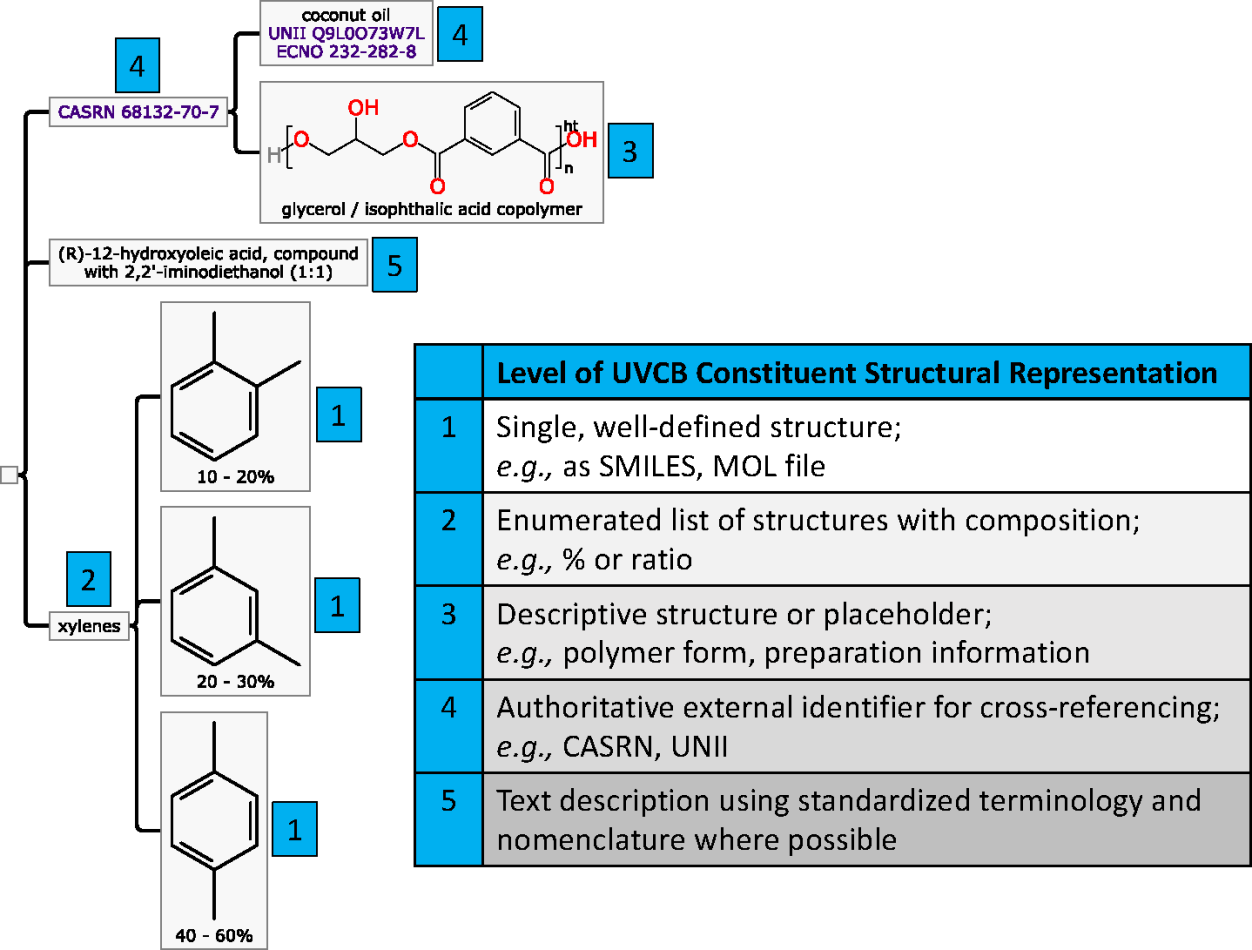
(left) Graphical illustration of the proposed UVCB data structure expressing constituents, concentrations/composition, and hierarchy, shown representing a “mixture of ‘coconut oil, polymer with glycerol and isophthalic acid’ (CASRN 68132-70-7) and ‘(*R*)-12-hydoxyoleic acid, compound with 2,2′-iminodiethanol (1:1)’ (CASRN 94232-00-5) dissolved in xylenes (CASRN 1330-20-7)” for demonstrative purposes.^90^ (bottom right) Different specificity levels of available information on UVCB constituent structural representation, in decreasing order of preference from 1 to 5.

For any given constituent in a mixture hierarchy, the specificity of constituent structural information available can be roughly characterized into five levels that indicate what types of cheminformatics functions can be applied (Figure 3).

Ideally, sufficiently characterized UVCBs have enough associated structural information to achieve level 1 and/or level 2 for individual constituents. With a single, well-defined structure (level 1), almost all structure-related derived properties can be calculated: names and identifiers via algorithms; database identity via lookup; and numerous search types, e.g., structure equivalence, similarity, substructure. Most importantly for chemical assessment, prediction of physicochemical, degradation, and (eco)toxicological properties via quantitative structure–activity relationships becomes possible. The same is generally true for level 2, but it is only viable up to the point when enumerating all isomers/congeners/homologues is practical.

Level 3 captures the essence of the UVCB problem: there is something known *about* the chemical entities present but this information often cannot be readily converted into a manageable set of discrete constituents. For poorly defined constituents, chemical information is often reported in a form accessible to the experimentalist to a certain extent,^46^ such as classes of chemical functionality (e.g., a form of starch is known to contain carbohydrate substructures), an industrial mixture described as the reaction products of certain input structures, polymers that may be indicated by providing the repeating units, and a constituent that may be described as all of the molecules from a source which distilled within a certain temperature range. The information known to the creator of the UVCB entry is sometimes only sufficient to enumerate a representative selection of molecules, but even when it is not, there might still be possibilities to narrow down what the molecules could be (e.g., by considering typical outputs from a given reaction type) and, subsequently, the appropriate queries and comparisons.

UVCBs may contain constituents that are defined in some sense other than chemical characteristics, which is commonly the case when using biologically sourced materials, corresponding to level 4. Many materials have an officially defined provenance and can be linked to a formal description using an identifier maintained or used by an authoritative organization, e.g., CASRN^177^ or International Nomenclature Cosmetic Ingredient names.^91^ These identifiers may be traced to the primary literature or preparation description (e.g., how to extract a fraction from a plant grown under certain conditions), but often they do not always provide meaningful, unambiguous chemical information, as discussed elsewhere.^46^

The final fallback, level 5, is to provide a text description of the substance, which facilitates keyword searching but is likely only understandable by domain experts. Very few higher-order text analyses are possible with current methods. However, such text-based fields could be supported by the development of ontologies or standardized terms (e.g., “acetylated”, “sulfurized”, or examples from European Union guidance^42^) that have formal definitions and should be used consistently by all stakeholders.

The above scheme is intended to be applicable to all UVCBs as a means of systematizing whatever information is currently available albeit possibly incomplete, for quality control of future reporting and to guide future characterization initiatives. Overall, but especially for chemicals assessment, levels 1 and 2 represent the most desirable levels of detail and should ideally be reflected in corresponding substance registration and characterization efforts.

## 3 Hazard Assessment of UVCBs

Different regulatory approaches exist around the world concerning the hazard assessment of UVCBs, some of which were reviewed elsewhere.^62^ In the United States, the EPA has not issued any guidelines specifically addressing UVCB testing and instead relies on a case-by-case approach.^29,62^ In Canada, UVCBs were prioritized^92^ within the ecological risk classification approach under the Chemicals Management Plan^93^ and assessed case by case using a weight of evidence approach (section 3.3.1), typically within chemical class specific groups, e.g., quaternary ammonium compounds, resins and rosins, etc. The groupings were identified on the basis of structural or functional similarities and were chosen according to several factors related to assessment efficiencies and avoiding regrettable substitution, among others. Alternative grouping strategies by common fate properties and ecotoxicological effects have also been recommended^62,82^ and performed based on common biological activity signatures,^68^ toxicological and biodegradability end points,^49^ and industrial use/emission patterns (section 4.1). Under the European Union’s REACH framework, certain hazard information must be provided with all registered UVCBs depending on the registration tonnage band and uses.^94^ Multiple UVCBs have been assessed under the Australian Inventory Multitiered Assessment and Prioritisation Tier 1 framework,^95^ but there is no specific UVCB guidance. Overall, UVCBs present challenges to regulatory frameworks concerning hazard assessment and communication, with specific issues related to testing strategies.

### 3.1 Overarching Hazard Classification and Communication: GHS

A primary outcome of hazard assessment is hazard classification, e.g., following the conventions of the Globally Harmonized System of Classification and Labeling (GHS). There is still no specific official guidance on UVCBs in the latest (ninth) revision of the GHS,^96^ despite early initiatives to develop GHS guidance for petroleum UVCBs,^97^ though a whole-mixture toxicity assessment is recommended for hazard classification of environmental and human health hazards and skin corrosion/irritation as well as for whole-mixture environmental biodegradation.^96^ If only part of the mixture is known, a suite of bridging principles can be applied to predict the mixture classification. However, explicit guidance for mixtures exists. Applying GHS guidance for mixtures requires knowledge of all constituents present so that all the respective hazards can be evaluated, which may be possible for certain UVCBs. For example, the MeClas tool, used for hazard identification and classification, assumes all metal constituents are known in complex inorganic UVCBs.^98^ Similarly, an adapted implementation of GHS was proposed for petroleum UVCBs,^99^ where petroleum streams are considered unique substances each having individual CASRNs, which can be sorted into categories based on similar physicochemical/toxicological profiles and then evaluated for hazard accordingly. Implementing this same method for hydrocarbon solvents has been deemed feasible by Mckee et al.^100^ However, for most other types of UVCBs, detailed knowledge of constituents may not be available, thus limiting the applicability of current GHS mixtures guidance to UVCBs because GHS requires all constituents to be known.

Despite the lack of UVCB-specific GHS guidance, testing strategies for hazard classification of UVCBs are under development.^101^ Moreover, there is some evidence of partial GHS classification of certain UVCBs such as “Juniper, *Juniperus virginiana*” (CASRN 85085-41-2);^178^ however, it is not clear how such classification was achieved, further supporting the need for specific transparent guidance for classifying UVCBs under GHS. In future guidance, some element to encode uncertainty could be introduced, e.g., as pictograms/classification/hazard statements to reflect uncertainty or incomplete understanding of the given UVCB composition and thus hazards.

### 3.2 General Approaches to Assess Persistence, Bioaccumulation, and Toxicity (PBT)

**Figure 4:**
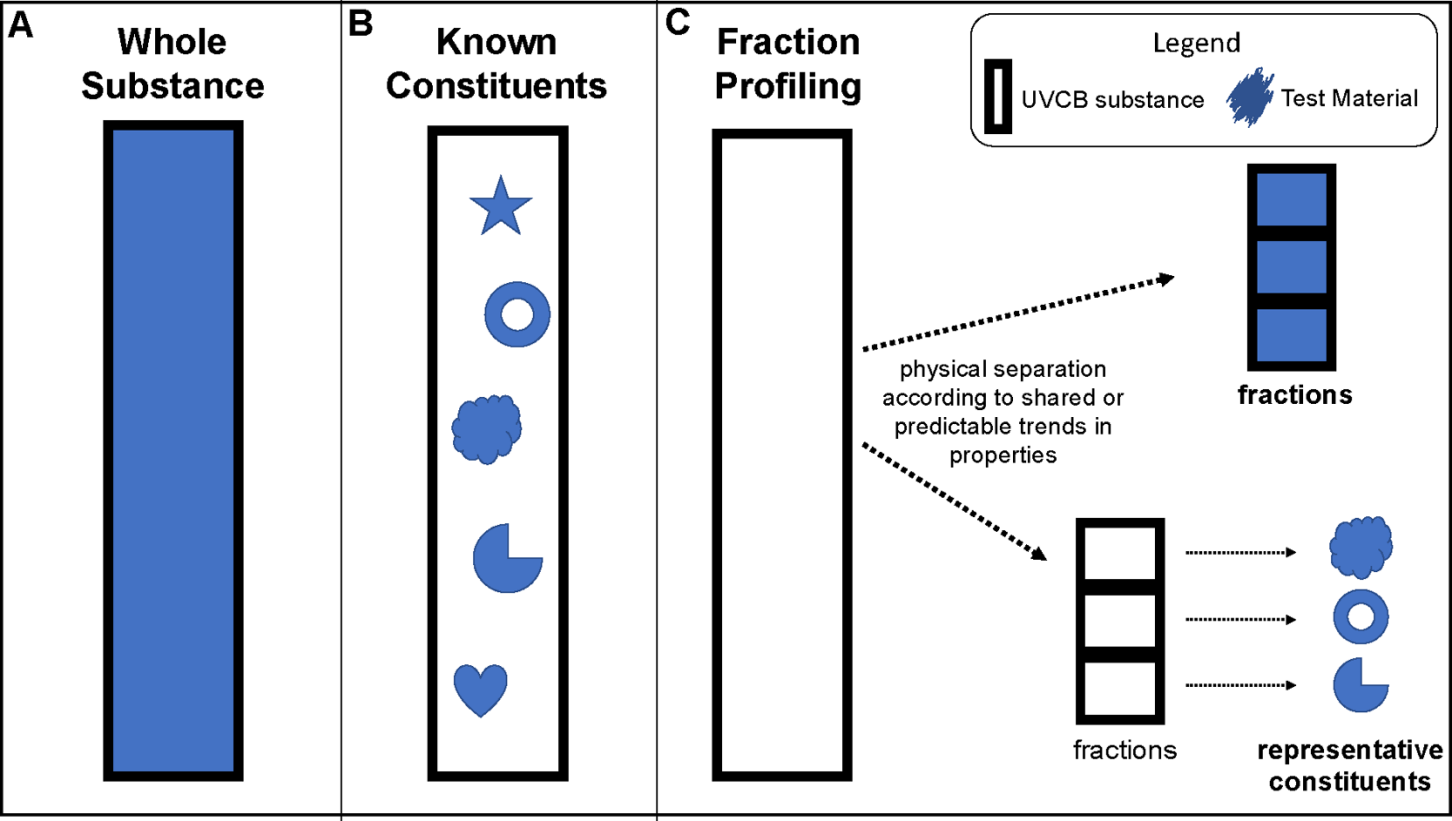
Schematic representation of the three main experimental approaches prescribed for PBT assessment of UVCB substances.^62,102^

Three main approaches have been prescribed for empirical testing of P, B, and/or T properties of UVCBs:^62,102^ whole substance, known constituents, and fraction profiling (Figure 4). The European Union’s REACH encourages a combination thereof where necessary, for example, when knowledge of the substance evolves during assessment or if tested constituents are sufficiently different from the remaining composition of the substance.^102^ In the whole-substance approach, the entire UVCB undergoes testing and assessment (Figure 4A). However, because of substance complexity and potentially variable constituent solubilities that can cause challenging test conditions for the whole-substance approach, the known-constituent approach may be favored (Figure 4B). Known constituents can represent the entire UVCB in testing and assessment if they can be isolated, are present at relevant concentrations within the substance, and represent worst-case characteristics. Alternatively, the fraction-profiling approach involves splitting the whole substance into so-called “fractions”, and either the fractions themselves or representative constituent(s) of each fraction are tested (Figure 4C). The latter is also known as the “block method”. Physical separation of the whole substance into fractions is performed such that constituents within each fraction show a predictable trend in properties, e.g., physicochemical, structural, mode of action (MoA), and degradation.^62,102^ Read across is expected to be applicable within the constituents of a given fraction.^102^ The hydrocarbon block method (HBM)^82^ is a specific form of fraction profiling for petroleum UVCBs and, together with its associated assessment tools (e.g., PetroTox,^103^ a spreadsheet model designed to calculate the toxicity of petroleum products to aquatic organisms), has been the result of 30 years of work in the petroleum sector. In the first EU Technical Guidance Document, HBM was prescribed for assessing environmental risks of petroleum substances.^104^

Detailed discussions of the advantages and disadvantages of each approach are available elsewhere.^62,102^ Briefly, testing whole substances does not require generation of new test material, but results may not be representative of all constituents; known constituents are relatively easy to test as they are discrete and well-characterized but may require more effort to characterize up front and may not ultimately be representative of the whole substance; and fraction profiling allows more targeted assessment than whole substance but requires generation of test material, i.e., the fractions.

A fourth, less common approach consists of *in silico* PBT screening, as recently performed for 884 constituents in the same hydrocarbon block of alkylated three-ring PAHs via relative trend analysis of experimental and modeled data.^105^ The half-lives of petroleum products modeled by BioHCWin were validated by newly generated empirical data, suggesting that preliminary persistence screening of petroleum UVCBs is feasible using models.^106^ Although *in silico* PBT screening may circumvent experimental difficulties associated with dealing with complex UVCBs, it ultimately requires experimental validation, is extremely data-intensive, and thus is only viable for well-studied UVCBs whose constituents are well-characterized and chemically similar.

The availability of PBT-related studies for a given UVCB is highly dependent on the nature of the substance itself and factors such as the substance’s practical applications, economic/industrial importance, availability of reference material, and overall environmental relevance. For example, there has been relatively more research on the degradation, bioaccumulation, and toxicity behaviors of petroleum substances and chlorinated paraffins,^23^ as reflected in extant prioritization schemes for PBT assessment, likely because these are well-known UVCBs.^107^ In comparison, there is little knowledge of the PBT characteristics of lesser-known UVCBs such as “Morpholine, 4-C_12–14_-alkyl derivs.” (CASRN 1402434-48-3), “Alcohols, lanolin” (CASRN 8027-33-6), or “Fatty acids C_18_ unsat, reaction products with pentaethylenehexamine” (CASRN 1224966-13-5).

#### 3.2.1 Persistence

Generally, ISO- and OECD-standardized tests for degradability were originally developed for fully characterized substances and by default adopt a whole-substance approach. The biodegradation screening tests, e.g., ready biodegradability (OECD 301A–301F) and inherent degradability (OECD 302A–302F), typically measure CO_2_ formation, theoretical oxygen demand, or substrate decay. These methods can be applied to UVCBs, although these screening tests may not accurately reflect whole-substance persistence. The simulation biodegradation tests in soil, sediment, and surface water (OECD 304, 307, 308, 309) require ^14^C labeled compounds to quantify loss of the parent and identify transformation products. While these are more challenging to perform for UVCBs, efforts involving, e.g., fully labeled chlorinated paraffin mixtures already exist.^108^

Screening tests based on CO_2_ formation or oxygen demand quantification can be applied to UVCBs, but it is possible that the persistence of a whole UVCB could be incorrectly determined by assessing its more degradable constituents, despite the UVCB containing persistent constituents. As these tests do not provide detailed persistence information at the constituent level, the true degradability of a UVCB can be subject to interpretation and may have to be evaluated on a case-to-case basis.^62^ An alternative measure for testing a UVCB’s ready biodegradability has been proposed, where a carbon balance approach is used to derive the level of ultimately transformed organic carbon (sum of mineralized carbon and carbon converted to biomass) in aerobic biodegradation tests as a measure of ready biodegradability, but it may be limited to only substances whose carbon content can be measured.^109^

In certain cases where the UVCB has a relatively simple chemical composition, it may be justifiable to apply bulk degradation test results to the entire UVCB substance. For example gas-to-liquid synthetic hydrocarbons were deemed “sufficiently homologous”, such that nonspecific results from ready biodegradability tests “can be used to conclude on their biodegradability as a whole”.^110^ Alternatively, if tested known constituents cover an appropriately broad and relevant chemical space that would account for substance variability, degradation results could be extrapolated to other substances within that applicability domain, as performed with kinetic studies of test chemicals commonly found in petroleum substances.^111,112^

Overall, evaluating UVCB persistence is still in the method development stage, as there are many technical and analytical challenges, e.g., possible impact of mixture effects (where certain constituents may enhance or diminish the biodegradation kinetics of other constituents present), for which whole-substance testing is necessary to evaluate.^71,113,114^ An important outcome of these works for informing future studies is that test concentrations should be kept at low, environmentally relevant concentrations to avoid mixture toxicity affecting biodegradation kinetics. To date, most studies focused on developing persistence tests for hydrophobic UVCBs. Testing strategies for UVCBs with other types of challenging physicochemical properties (e.g., hydrophilic, volatile) should be developed to enable the persistence testing of UVCBs with different properties.

#### 3.2.2 Bioaccumulation

Initial bioaccumulation screening relies on the octanol–water partition coefficient (*K*_ow_), but as with persistence testing, different constituents may have different *K*_ow_ values and thus different bioaccumulative properties that could complicate results interpretation for whole UVCBs. Initial estimates of whole-substance bioaccumulation potential could be inductively concluded if analytical methods such as high performance liquid chromatography capable of capturing multiple constituents indicate whether all constituents either exceed or are below the common regulatory log *K*_ow_ 4.5 threshold for screening bioaccumulation assessment.^102^ However, as equilibrium partitioning may not be the only process determining bioaccumulation, log *K*_ow_ > 4.5 does not imply that a chemical is bioaccumulative, but further evaluations are required. In the case of UVCBs, different constituents may undergo active uptake, metabolism, and/or excretion to varying extents.^29^ The recommended approach^62^ has been to consider the bioaccumulative properties of a UVCB’s representative/main constituents instead of those of the whole substance itself. Bioconcentration factors (BCFs) were successfully determined for the main constituents of “cedarwood Virginia oil” (CASRN 8000-27-9) in rainbow trout this way,^115^ and continued work by the same authors developed an analytical technique within a suspect-screening approach that circumvents the need to have *a priori* knowledge of constituent identities and available analytical standards.^116^ Several technical substance mixtures of chlorinated paraffins, typically already subdivided according to chain length, were found to be bioaccumulative in *Daphnia magna*.^117^

An extended discussion of measuring UVCB bioaccumulation is available elsewhere.^29^ Overall, there are very few bioaccumulation studies of UVCBs and their constituents, and more work is needed to develop methods for future bioaccumulation studies of other UVCBs, such as testing the suitability of *in vitro* methods.^29^

#### 3.2.3 Toxicity

Toxicity assessment requires aquatic toxicity testing and/or the evaluation if the substance poses a human health hazard, namely if it is carcinogenic, mutagenic, or reproduction toxic (CMR), an endocrine disrupting compound (EDC), or mediates specific target organ toxicity (STOT). Aquatic toxicity testing of UVCBs is challenging from two perspectives. First, it involves the ability to correctly define the dose of the substance and make sure a constant test concentration is maintained over the testing period. Second, the constituents of many UVCBs can be very hydrophobic, making dosing challenging even for single compounds. Toxicity is mediated by bioavailability, which is limited by solubility and the sample preparation methods used. Interestingly, very hydrophobic chemicals are of such low solubility that toxic concentrations cannot be achieved for single compounds but can be achieved for mixtures.^118^ As UVCBs have multiple constituents of likely varying solubilities and percentage compositions, aquatic toxicity testing of UVCBs poses technical challenges for hydrophobic and/or volatile constituents. Thus, considerable studies in recent years have focused on developing improved toxicity testing methodologies for UVCBs, especially with respect to dosing of volatile, hydrophobic, and volatile and hydrophobic UVCBs,^119−121^ as well as analyzing the effect of sample preparation on bioavailability.^122^

Overall, modeling toxicity and testing of UVCBs have mostly focused on petroleum substances,^119−121,123^ solvents,^84,124^ and chlorinated paraffins.^23,125−127^ Future method development and toxicity evaluations of other UVCBs are warranted.

### 3.3 Additional Considerations for Comprehensive Effect Assessment

Exposure to a UVCB substance results in combined exposure to more than one chemical at the same time. Therefore, from a chemical and toxicological perspective, UVCBs *are* mixtures despite the legal distinction drawn between UVCBs and mixtures within regulatory frameworks.^17,128,129^ Thus, for the purposes of comprehensive effect assessment, the same established principles for assessing mixture toxicity are applicable to assessing UVCBs.^130^

#### 3.3.1 Whole-Mixture Testing

Comprehensive effect assessment requires a whole-substance approach where the effect of the mixture is tested. In principle, dosing mixtures into bioassays follows the sample principles as for single chemicals, and since solubility of each compound is additive in a mixture, overall, more chemicals can be brought into solution in the case of UVCBs as compared to single chemicals. However, there are challenges because the mixture composition must not be changed since the exposure concentrations of mixtures cannot be confirmed analytically.

Dosing remains a particular challenge for UVCBs that contain many low solubility components because the solubility of whole mixtures depends on the solubility of the least soluble constituent during aquatic toxicity testing. Therefore, there is a danger that the more hydrophobic chemicals are not dissolved and hence not bioavailable, and the effect is dominated by the more soluble constituent. As more hydrophobic chemicals are typically more potent than more hydrophilic chemicals, this may lead to dramatic underestimation of toxicity.

Another complication is UVCBs with volatile components or volatile and hydrophobic components. For such UVCBs, the water accommodated fraction (WAF) approach is intended as a “last resort” if all other means of ensuring stable substance concentrations during testing have been exhausted,^102^ or as an “additional supporting line of evidence” to empirical and modeled data.^131^ It involves expressing aquatic toxicity in terms of loading rate (ratio of test substance to aqueous medium used to make the aquatic toxicity test medium), thereby providing a measure of relative toxicity at concentrations equating to the apparent solubility of each component and not their actual abundance in the mixture. However, WAF has fundamental drawbacks: it represents only a fraction and not the whole substance (whose chemical identity is subject to uncertainty), mixture composition may be altered compared to the UVCB it is prepared from, and the WAF composition depends on preparation techniques. Issues related to WAF results interpretation for coal tar pitch and kerosene/jet fuel UVCBs within regulatory processes of the U.S. EPA and REACH have been reported.^132^ Alternatives to WAF include solvent extraction followed by solvent spiking, generator systems, saturator columns, and passive dosing methods, the last of which has been in active development in recent years with respect to UVCBs.^119−121^

On balance, results from whole-mixture testing could be integrated into a weight of evidence (WoE) approach for UVCB assessment. In Canada’s WoE approach, multiple lines of evidence are considered in the assessment of a UVCB: for example, besides considering WAF test results, other aspects such as representative structures, individual constituent toxicity, and additive toxicity may also be evaluated together when deciding on a substance’s toxicity and capacity to cause adverse effects in the environment.

#### 3.3.2 Mixture Toxicity Models: Toxic Equivalence Approach for UVCBs

Ideally, choosing an appropriate mixture toxicity model for a given UVCB would be determined by knowledge of its constituents and composition. For example, UVCBs containing chemically diverse constituents with different MoAs would follow an independent action (IA) model of toxicity, while those with the same MoA would follow concentration addition (CA), whereas mixtures with known interactions between their constituents might cause synergistic or antagonistic effects. However, these effects are rare and typically happen in mixtures with few components and for highly specifically acting compounds such as in pesticide formulations;^133,134^ therefore synergism and antagonism are unlikely for UVCBs.

The simple CA model can be applied to UVCBs with relatively simple compositions and chemically similar constituents (e.g., UVCBs such as “Alcohols, C_9_–C_11_”). Even independently acting compounds often have mixture predictions very similar to CA or converge to the same mathematical model at low effect levels (<10%).^135,136^ Very complex UVCBs with many diverse constituents, albeit each individually present at very low concentrations below effect levels (e.g., petroleum, or biological materials like essential oils), would also follow CA. Provided that relative effect potencies (REPs) are independent of effect level or concentration in these cases, a toxic equivalency approach can be applied.^137^

The toxic equivalent concentration (TEQ) of a UVCB or any chemical mixture is the sum of the products of the concentration of each constituent *i* and its respective toxic equivalency factor (TEF_*i*_), where TEF_*i*_ is defined as the ratio of the effect of a reference compound to the effect of the constituent *i*. Such a reference compound could be a known representative constituent. TEFs are consensus values for dioxin-like chemicals,^138^ but a conceptually and mathematically identical approach could be taken using REP_*i*_’s from the same toxicity test^137^ (eq 1), where *C*_*i*_ is the concentration of constituent *i* in the mixture, EC_*i*_ is its effect concentration in the given bioassay, and EC_ref_ is the effect concentration of the reference compound.
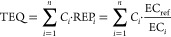
1

The TEQ approach was mentioned in the official European Union opinion on mixtures^139^ but no practical examples for UVCBs exist in the public domain as of yet. Currently, the whole-mixture approach is recommended in regulatory risk assessment of mixtures.^35^ In practice, if not all the EC_*i*_ values of the mixture components are known, they can be approximated by similar constituents, as was successfully demonstrated for the human health risk assessment of brominated flame retardant mixtures.^140^

In multiconstituent mixtures, not only does CA likely apply, but toxicity of complex mixtures is often reduced to baseline toxicity,^141^ which is the minimum toxicity triggered by nonspecific interactions of chemicals with biological membranes leading to disturbance of structure and functioning of cell and organelle membranes.^142^ Since all chemicals are equipotent with respect to baseline toxicity if effects are expressed as internal concentrations, there is a critical molar membrane burden above which effects can be observed. This level is around 200–500 mmol/kg lipid for LC_50_ of aquatic animals.^142−144^ The chemical properties of the chemicals decide only how much is taken up by the organism and ultimately distributed into the membranes, but once they are in the membrane all chemicals act close to equipotent. This means that critical membrane burdens or, for all practical matters, critical or lethal body burdens can easily be applied to mixtures.^145^ This principle has also been extended to mixtures in the so-called target lipid model (TLM).^146,147^

## 4 Exposure and Risk Assessment of UVCBs

### 4.1 Exposure Assessment

Within regulatory frameworks, exposure assessment of UVCBs is not always considered necessary and is highly dependent on the framework in question. For example, in the European Union, the outcomes of initial hazard assessments may already be enough to initiate risk management measures without having to assess UVCB exposure. However, in many other jurisdictions such as the United States, Canada, and Australia, a full risk assessment of chemical substances that includes exposure assessment is generally required to determine whether risk management measures should be triggered.

In cases where exposure assessment of UVCBs is necessary, regulators must deal with multiple challenging aspects of UVCB exposure, particularly with regard to environmental monitoring and biomonitoring. First, it is difficult to measure UVCBs in the environment because of their multiconstituent nature. Environmental monitoring typically only tracks single compounds, but because UVCBs comprise multiple constituents, validation issues may arise as it is difficult to attribute the detection of a particular constituent to the emission of a UVCB containing that constituent. Furthermore, environmental transformations of these constituents and potentially different fate and transport properties resulting in different exposure pathways could complicate this attribution further.^19,29^ Therefore, ideally full knowledge of constituent identities and compositions is needed for exposure assessment of UVCBs. However, as this has been difficult to achieve in practice, refining exposure scenarios by, e.g., considering the magnitude of emissions and current mitigation measures in place may help prioritize substance characterization efforts (section 2) needed for exposure assessment. Overall, some uncertainty will remain regarding unknown constituents and their unknown environmental fate and exposure properties, which is challenging to capture in the overall exposure assessment. It is important to convey this gap in knowledge/uncertainty as part of assessment outcomes.

Concepts for exposure assessment and fate and transport modeling of UVCBs are currently under active development.^29^ A review of publicly available electronic registration dossiers and risk assessment reports revealed three main approaches for estimating exposures of UVCBs: whole substance (section 4.1.1), constituent (section 4.1.2), and expert judgment (section 4.1.3).

#### 4.1.1 Whole-Substance Approach

UVCBs whose constituents are not clearly defined or are too complex in composition can be assessed as a whole. Relevant information such as import and manufacturing volumes, consumer uses, product use scenarios, and percent concentration within products are considered. An example is the assessment of the organic anthraquinone UVCB “9,10-Anthracenedione, 1,4-diamino-, N,N′-mixed 2-ethylhexyl and Me and pentyl derivs.” (CASRN 74499-36-8) by the Government of Canada (GoC) using the ConsExpo model to estimate oral and dermal exposures.^148^

Whole-substance exposure assessment can also be performed for groups of substances within, e.g., a common sector of industrial activity, as their exposures are considered very similar or identical. GoC assessed 57 sector-specific inorganic UVCBs used in metals, paper, and cement processing and manufacturing in this way.^149^ Exposure potential was evaluated on the basis of the status of the substance (e.g., “waste”, “byproduct”), and whether there were any preexisting measures to limit environmental exposure. In this example, exposure was emphasized over hazard in the overall characterization of risk, and as exposure was deemed negligible, regardless of hazard, risks to human health were considered low and harm to the environment not expected. However, such grouping and disproportionate emphasis on exposure over hazard could be detrimental for substances with specific MoAs and/or high toxicity, uncertainties in assessing exposure potential persist, and there may be caveats in assuming the preexisting measures to limit exposure were adequate.

#### 4.1.2 Constituent Approach

Each constituent and/or representative constituents must be known and should undergo individual exposure assessment (or the relevant information gathered from the literature) before the assessments are combined to give an overall exposure assessment of the UVCB. This approach has been recommended for inorganic UVCBs, where assessing constituents would be similar to “standard metal exposure assessment” and should take into account speciation behavior, assuming the worst-case scenario where information is incomplete.^150^ In the final combination step of the parallel constituent assessments, multidimensional risk characterization ratio tables (constituent × exposure route × local/systemic effects, short term/long term) are generated.^150^ Examples exist under the EU REACH, e.g., the inorganic UVCB “Lead alloy, base, Pb, Sn, dross” (CASRN 69011-60-5), whose dossier states “assessing transport and distribution of the UVCB substance has no meaning”, as the “metals contained in the UVCB have been assessed in the respective risk assessments”.^151^

#### 4.1.3 Expert Judgment

Expert judgment can be used where there is insufficient knowledge of hazard and exposure and no representative structure(s) to describe the substance. Qualitative exposure classification was performed for 192 organic UVCBs^152^ and an anthraquinone UVCB (CASRN 74499-36-8) by GoC.^148^ Supporting information, e.g., industry surveys and consideration of similar substances, was also taken into account. However, more information is needed to transparently illustrate how these expert judgments are carried out and validated, and to assess whether such judgments can be automated in the future.

### 4.2 Risk Assessment of UVCBs

Risk characterization traditionally involves the calculation of a risk quotient: the outcome of exposure assessment (e.g., predicted environmental concentration, PEC) is divided by that of effect assessment (e.g., predicted no effect concentration, PNEC). Risk quotients of individual components of a mixture are additive to yield the risk index (RI) if CA applies for the mixture effect (eq 2).
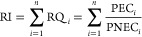
2Hence for mixtures and therefore also for UVCBs, one could calculate the TEQ as described above and use that in relation to the PNEC of the reference compounds used to derive the TEQ (eq 3).
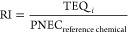
3

Comprehensive environmental risk assessments including both effect and exposure of whole substances have been developed for two particular types of UVCBs: petroleum products (PETRORISK)^153^ and hydrocarbon solvents.^154^ While substance complexity and variability are reflected in hazard and risk predictions by PETRORISK,^155^ careful ongoing evaluation of these models is necessary, as PETRORISK was found to underestimate the environmental risks of petroleum use and production.^156^ Methods for other UVCBs have yet to be established.

### 4.3 Current Regulatory Activities, Perspectives, and Priorities

Overall, many regulatory authorities have endeavored over the past decade to develop scientifically sound and consistent approaches for the assessment of UVCBs. However, the availability of specific (standardized) guidance to achieve this is still limited to date. In practice, both whole-substance^131,148,149,157^ and constituent-based^151^ approaches are being used in current regulatory assessments, informed by established principles such as those of HBM (but tailored to suit chemistries other than petroleum), as well as guidance on mixtures.^158−161^ Given the large range in complexity, chemical classes, and data availability for UVCBs, it is not always possible to be prescriptive for all aspects of hazard, exposure, and/or risk assessment. Therefore, a case-by-case approach is still the preferred and potentially only viable approach for certain UVCBs, but it may pose a burden for risk assessors and result in less predictability for stakeholders.

## 5 Discussion: Challenges and Opportunities

Several systemic factors contribute to the challenges posed by UVCBs: information gaps in chemical identities and compositions stemming from the registration process, inadequate chemical representation and nomenclature hindering identification and database searchability, lack of analytical standards and methods tailored specifically to UVCBs, challenging conditions for PBT testing, and the sheer number of UVCBs to be assessed. Below, key opportunities and steps forward in addressing these challenges are summarized.

### 5.1 Registration

Fundamental knowledge gaps in UVCB identities could be avoided from the start if information requirements to register UVCBs were increased, in tandem with implementing better methods for chemical representation. Requiring machine-readable structural information, including representative or generic structures for constituents, *and* compliance and quality checks during registration may assist with this. Standardized description terminology should be developed toward improving UVCB nomenclature for registration, possibly with the support of IUPAC and CAS. Potential avenues to implement these information types include GHS, OECD, and IUCLID.

### 5.2 Chemical Representation and Information Management

Chemical representation issues linked to nomenclature, structure, and use of closed identifiers such as CASRN still hinder precise identification of UVCBs. Machine-readable representations to enable unambiguous substance identification and searchability such as G SMILES and the open MInChI represent possible solutions. Future initiatives to improve chemical representation of UVCBs could be spearheaded by organizations such as IUPAC’s InChI Subcommittee focusing on capturing mixture composition using MInChI.^162^

UVCB information must be better organized to enable (1) capture of their multiconstituent and multifaceted properties, (2) quality checks, and (3) detection of information gaps. A hierarchical data format and associated constituent representation scheme were proposed to achieve this (Figure 3). It is important for stakeholders to consider this format in further discussions toward achieving a standardized system so that future reporting, storing, and exchanging of UVCB information become more accurate and precise. Future research in this area such as proofs of concept and analyses on how our proposed format could function for several types of UVCBs is highly anticipated.

### 5.3 UVCB Characterization: Toward Environmental Detection and Monitoring

UVCB characterization is currently achieved by two means: cheminformatics and analytical chemistry. Cheminformatics methods rely on text parsing and cross-linking information that already exists in databases and, because these are often done *in silico*, are potentially the fastest and most scalable characterization approach. However, these methods are fundamentally limited by the availability and quality^61^ of preexisting UVCB information in the public domain.

Ultimately, analytical characterization will be necessary to generate (new) knowledge on UVCB identities and compositions. UVCBs other than petroleum substances warrant characterization, particularly if they are high production, toxic, or heavily emitted into the environment. Given their complex and unknown characteristics, nontarget strategies^163,164^ involving multiple analytical techniques to give complementary information will be required to elucidate UVCBs, especially as they may have generic elemental compositions (e.g., only C, H, O, N) and molecular formulas similar to hundreds of natural products, making them hard to distinguish from environmental matrices. Chemometrics or cheminformatics tools could be used for prioritization based on substructure or toxicity.^165,166^

Overall, UVCB characterization is a prospective area of dynamic research, especially as knowledge of their identities becomes indispensable for answering “bigger questions” such as investigating known toxicity end points associated with constituents requiring identification. Successful characterization efforts and analytical method development contributing to better knowledge of UVCB identities will likely open more avenues for their environmental detection and monitoring. Chlorinated paraffins^23,73,167^ are a good example, as their constituents are known and have distinctive analytical signatures (e.g., homology, multiple halogens present) which facilitate identification.^168−170^ However, for UVCBs with very different constituents, new concepts and analytical methods for their environmental detection will be necessary. Several open questions remain, such as how many constituents must be co-detected to conclude on the detection of a specific UVCB, how potential transformations^171^ and partitioning of different constituents across multiple environmental compartments can be accounted for, etc.

### 5.4 Hazard, Exposure, and Risk Assessment

Existing testing strategies for single-compound end-point assessments should be adapted to the multiconstituent characteristics of UVCBs following one of three approaches: whole substance, known constituent, and fraction profiling. Standardized testing methods are needed, requiring cooperation among the relevant stakeholders to develop them. Strategies such as grouping and read across may help streamline chemicals assessment, especially for UVCBs with similar constituents or properties, as would applying appropriate mixture toxicity models (i.e., CA and/or TEQ) for comprehensive effect assessment in a complementary approach to further substance characterization.

To support chemicals assessment of UVCBs, current priorities for future research and action include the following: (1) improving the quality and availability of information on UVCB components, (2) deepening the understanding of manufacturing and use practices and the release potential of UVCBs to the environment, (3) developing tools to estimate exposure of multiconstituent substances in environmental matrices and biota, (4) developing standard hazard and fate test and assessment methods for UVCBs, and (5) improving approaches to communicating complex risk assessment findings to stakeholders.

Concerted efforts from all stakeholders are needed to systematically address UVCBs, particularly in identifying and managing those that present unacceptable risks. There are tens of thousands of UVCBs on the market, and risk assessment prioritization schemes such as those available for petroleum substances^107^ should be devised for other UVCBs based on, e.g., detection in the environment, highest production volumes, and known toxicity and/or exposures (preliminary initiatives within NORMAN Network activities are underway^172^). Meanwhile, stakeholders may also consider simplification^78^ and sustainable circular use^173^ principles of UVCBs toward their sound management in the medium to long term.

## Supporting Information Available

Details of individual UVCBs mentioned in this review (TXT)
Summary table of major databases and inventories containing UVCBs (PDF)
