# Supplementary material for: The Next Frontier of Environmental Unknowns: Substances of Unknown or Variable Composition, Complex Reaction Products, or Biological Materials (UVCBs)
Source: Environ Sci Technol. 2022 May 9;56(12):7448–66. doi: 10.1021/acs.est.2c00321 (PMC9228065; doi:10.1021/acs.est.2c00321)
Supplement: Supplementary file 2 — es2c00321_si_002.pdf [file es2c00321_si_002.pdf]

Supporting Information

The Next Frontier of Environmental Unknowns: Substances of Unknown or Variable Composition, Complex Reaction Products or Biological Materials (UVCBs)

Adelene Lai[1,2*], Alex M. Clark[3], Beate I. Escher[4,5], Marc Fernandez[6], Leah R. McEwen[7,8], Zhenyu Tian[9], Zhanyun Wang[10,11], and Emma L. Schymanski[1*]

ORCIDs: AL: 0000-0002-2985-6473, AMC: 0000-0002-3395-4666, BIE: 0000-0002-5304-706X, LRM: 0000-0003-2968-1674, ZT: 0000-0002-7491-7028, ZW: 0000-0001-9914-7659, ELS: 0000-0001-6868-8145

[1]Luxembourg Centre for Systems Biomedicine (LCSB), University of Luxembourg, 6 avenue du Swing, 4367 Belvaux, Luxembourg.

[2]Institute for Inorganic and Analytical Chemistry, Friedrich-Schiller University, Lessing Strasse 8, 07743 Jena, Germany.

[3]Collaborative Drug Discovery, Burlingame, CA, USA.

[4]Helmholtz Centre for Environmental Research GmbH – UFZ, Permoserstraße 15, 04318 Leipzig, Germany.

[5] Environmental Toxicology, Center for Applied Geosciences, Eberhard Karls University Tübingen, 72076 Tübingen, Germany.

[6]Environment and Climate Change Canada, Vancouver, British Columbia, Canada.

[7]Cornell University, Ithaca, NY, USA.

[8]International Union of Pure and Applied Chemistry, Research Triangle Park, NC, USA.

[9] Department of Chemistry and Chemical Biology, Department of Marine and Environmental Sciences, Northeastern University, Boston, 02115, MA, USA

[10]Chair of Ecological Systems Design, Institute of Environmental Engineering, ETH Zürich, 8093 Zürich, Switzerland.

[11] Empa − Swiss Federal Laboratories for Materials Science and Technology, Technology and Society Laboratory, Lerchenfeldstrasse 5, 9014 St. Gallen, Switzerland

Corresponding Authors

Pages: 3
Figures: 0
Tables:1

Table S2: Summary of major inventories/databases containing UVCBs and the relevant information fields within them (as of August 2021).

Inventory/ Database	Format	Substance Name Field	CASRN Field	Internal ID Field	Indicative UVCB flag	Chemical Structure Field	Physico-chemical Properties Field	Programmatic Accessibility	Notes
Non-confidential EPA TSCA Inventory[1]	Downloadable .csv or Microsoft Access database, version updates over time.	Y	Y	Y – ID	Y (16,988 of 68,167 are UVCBs, as of August 2021)	N	N	N	TCSA has 'chemical substance definition' field (DEF; publicly accessible) containing a short description of the substance *e.g.,* Castor oil, CAS 8001-79-4: *Extractives and their physically modified derivatives. It consists primarily of the glycerides of the fatty acid ricinoleic. (Ricinus communis).*
EPA CompTox Dashboard[2,3]	Online database	Y	Y	Y – DTXSID	N	Y (see Notes)	Y	N	CompTox Dashboard contains Synonyms field, Chemical Functional Use, Product & Use Categories, Production Volume, Bioactivity, and multiple links to literature.<br><br>Chemical Structures: 'Markush Children' and 'Constituents' included as 'Related Chemicals'
REACH Registered Substances[4]	Online database	Y	Y	Y – EC No.	N	Y (see Notes)	Y	N	ECHA database has Regulatory Process Names, IUPAC Names, Other Identifiers. Has information on Regulatory Context *i.e.,* if it appears in any regulatory lists. Has short description of substance: *e.g.,* CAS RN 8001-79-4, *extractives and their physically modified derivatives. It consists primarily of the glycerides of the fatty acid ricinoleic. (Ricinus communis, Euphorbiaceae).*<br>Chemical Structures: availability varies; can be represented as molecular formula, SMILES, InChI.
EC Inventory (includes EINECS + ELINCS + NLP)[5]	Online database or downloadable .csv	Y – EC and ECHA Name	Y	Y – EC No.	N	N	Y	N	EINECS and ELINCS contain short description of substance, *e.g.,* CAS RN 8001-79-4, "*Extractives and their physically modified derivatives. It consists primarily of the glycerides of the fatty acid ricinoleic. (Ricinus communis, Euphorbiaceae).*"
CAS Common Chemistry[6]	Online database	Y	Y	Y- CASRN	N	N	Y when available	N	"Other names", experimental properties, deleted and alternate CAS Registry Numbers
SciFinder[7]	Online database (proprietary)	Y	Y	Y-CASRN	Y	Y	Y when available	N	"Other names", experimental properties (more than Common Chemistry), regulatory information, deleted and alternate CAS Registry Numbers
PubChem[8,9]	Online database	Y	Y	Y- Substance SI	Y (see Notes)	Y	Y when available	Y	PubChem has depositor-supplied synonyms field. Indicative UVCB Flag: available if depositor comments include the term UVCB.

References

(1) United States Environmental Protection Agency. How to Access the TSCA Inventory. https://www.epa.gov/tsca-inventory/how-access-tsca-inventory (accessed 2021 -09 -05).

(2) United States Environmental Protection Agency. CompTox Portal. https://comptox.epa.gov/ (accessed 2021 -09 -05).

(3) Williams, A. J.; Grulke, C. M.; Edwards, J.; McEachran, A. D.; Mansouri, K.; Baker, N. C.; Patlewicz, G.; Shah, I.; Wambaugh, J. F.; Judson, R. S.; Richard, A. M. The CompTox Chemistry Dashboard: A Community Data Resource for Environmental Chemistry. *J Cheminform* **2017**, *9* (1), 61. https://doi.org/10.1186/s13321-017-0247-6.

(4) European Chemicals Agency. Advanced Search for Chemicals - ECHA. https://echa.europa.eu/advanced-search-for-chemicals?p_p_id=dissadvancedsearch_WAR_disssearchportlet&p_p_lifecycle=0 (accessed 2021 -09 -05).

(5) European Chemicals Agency. EC Inventory. https://echa.europa.eu/information-on-chemicals/ec-inventory (accessed 2021 -09 -05).

(6) Chemical Abstracts Service. CAS Common Chemistry. https://commonchemistry.cas.org/ (accessed 2021 -09 -05).

(7) Chemical Abstracts Service. SciFinder^{n}. https://scifinder.cas.org (accessed 2021 -09 -05).

(8) National Institutes of Health. PubChem. https://pubchem.ncbi.nlm.nih.gov/ (accessed 2020 -08 -13).

(9) Kim, S.; Chen, J.; Cheng, T.; Gindulyte, A.; He, J.; He, S.; Li, Q.; Shoemaker, B. A.; Thiessen, P. A.; Yu, B.; Zaslavsky, L.; Zhang, J.; Bolton, E. E. PubChem in 2021: New Data Content and Improved Web Interfaces. *Nucleic Acids Research* **2021**, *49* (D1), D1388–D1395. https://doi.org/10.1093/nar/gkaa971.
